# Multicenter consensus development of a checklist for lung injury prevention

**DOI:** 10.1186/cc11111

**Published:** 2012-03-20

**Authors:** JM Litell, O Gajic, J Sevransky, M Gong, DJ Murphy

**Affiliations:** 1 Mayo Clinic, Rochester, MN, USA; 2 Emory University School of Medicine, Atlanta, GA, USA; 3 Montefiore Medical Center, Bronx, NY, USA

## Introduction

Acute lung injury (ALI) is linked to almost 75,000 US deaths annually. The syndrome is defined clinically by criteria that identify only patients with established ALI, at which point treatment options are limited and largely supportive. After 40 years and more than 25 NIH-funded trials of ALI interventions, only supportive therapy with lung protective ventilation has been associated with a mortality benefit. The US Critical Illness and Injuries Trials Group lung injury prevention subgroup seeks to standardize best practices for patients at risk of ALI. The recently validated lung injury prediction score (LIPS) identifies patients at risk of ALI, and can prompt the early use of preventative interventions. This may attenuate the progression to ALI. This study seeks expert consensus about best practices in patients at risk of ALI, as determined by their LIPS. These practices will be incorporated into a checklist for lung injury prevention. Standardization of care may protect patients against ALI development and provide a uniform background for enrollment in other ALI trials.

## Methods

This study employed a Delphi selection process involving 38 intensivist participants using a web-based survey tool. In Round 1, participants were presented with 15 interventions proposed by investigators. Using a five-item Likert scale, they responded to the question: 'In your opinion as an expert, how sufficient is the evidence that this intervention reduces the risk of ALI in eligible patients?' Participants were also prompted to comment and submit additional items for consideration. In Round 2, participants followed the same approach to rate and comment on items submitted by the group. Finally, in Round 3, participants reviewed aggregated ratings and comments for all items, and voted for or against inclusion in the draft checklist. Inclusion was limited *a priori *to items with at least 70% agreement among participants.

## Results

Following Round 1, items submitted by participants were aggregated with minimal change into six additional items for Round 2. In Round 3, of the 21 total items, nine were endorsed by 70% of participants for inclusion in a draft checklist. These items were grouped conceptually into two domains: respiratory support and resuscitation.

## Conclusion

The Delphi process of expert consensus can be employed to develop a checklist of time-sensitive interventions, in a manner that combines available evidence with the perspective of expert clinicians.

